# Bilateral buccal space lipoma: A rare case presentation

**DOI:** 10.4317/jced.55568

**Published:** 2019-06-01

**Authors:** Lampros Goutzanis, Agamemnon Chliaoutakis, Demos Kalyvas

**Affiliations:** 1 Assistant Professor. Department of Oral and Maxillofacial Surgery, School of Dentistry, National and Kapodistrian University of Athens, Greece; 2 Post graduate Student. Department of Oral and Maxillofacial Surgery, School of Dentistry, National and Kapodistrian University of Athens, Greece; 3 Associate Professor. Department of Oral and Maxillofacial Surgery, School of Dentistry, National and Kapodistrian University of Athens, Greece

## Abstract

**Background:**

The lipoma of the oral cavity is a relatively rare pathology, characterized by overgrowth of the mature adipocytes. Buccal fat pad lipomas usually interfere with the esthetics and the function of the face.

**Case Report:**

A sixteen-year-old patient reported slow-growing swelling, which started two years ago. Clinical examination revealed two bilateral masses in the soft tissue. MRI imaging revealed a characteristic image of bilateral lipomas connected to the buccal fat pad. Surgical removal was conducted and the histology report confirmed our clinical diagnosis of common lipoma.

**Discussion:**

The lipoma of the buccal fat pad is a benign neoplasm of the adipose tissue. It should be removed when functional or esthetic problems occur and emphasis should be put on the correct surgical technique.

**Conclusions:**

The bilateral buccal fat pad lipoma is an extremely rare condition of the oral cavity. Surgical removal with intraoral approach is the preferable treatment, together with intense care of the anatomical structures of the buccal space.

** Key words:**Buccal space lipoma, oral cavity, buccal space, bilateral, buccal fat pad lipoma.

## Introduction

The lipoma is a benign mesenchymal neoplasm composed of mature adipocytes and usually surrounded by a fibrous capsule (1). Anatomically, the buccal fat pad is located within the compartment of the buccal space (2). The buccal fat pad is a tubular collection of adipose tissue that occupies a prominent position in the midface (2).

In the oral cavity, the lipoma is quite unusual, representing only 1-4% of benign tumors (1,3). Histologically, lipomas are categorized into common lipoma, fibrolipoma, osteolipoma, angiolipoma, spindle cell lipoma, myolipoma and chondroid lipoma (3). Clinically, the lipoma is described as a slow-growing, well-circumscribed mass of the soft tissue. The most common locations of lipoma in the oral cavity are the buccal mucosa, the tongue, the floor of the mouth, the vestibular area, the lower lip, the hard and soft palate and rarely the region of buccal fat pad (4). Our research of the recent literature (of the past ten years), in which the key words ‘buccal fat pad lipoma’ and ‘buccal space lipoma’ were used, showed plenty of oral cavity lipomas; however, only 5 cases of buccal fat pad lipoma were identified (4-7). By the literature review conducted, it is shown that the common lipoma of the buccal fat pad is an extremely rare neoplasm. Also, there is no reference of a bilateral lipoma in the literature.

## Case Report

A 16-year old female patient came at the university clinic, reporting bilateral swelling of the cheeks, especially on the right side. The patient had noticed that the swelling had started two years ago with no signs of pain, slowly growing and causing asymmetry of the face. The general medical history of the patient does not contain any condition.

**Figure 1:**
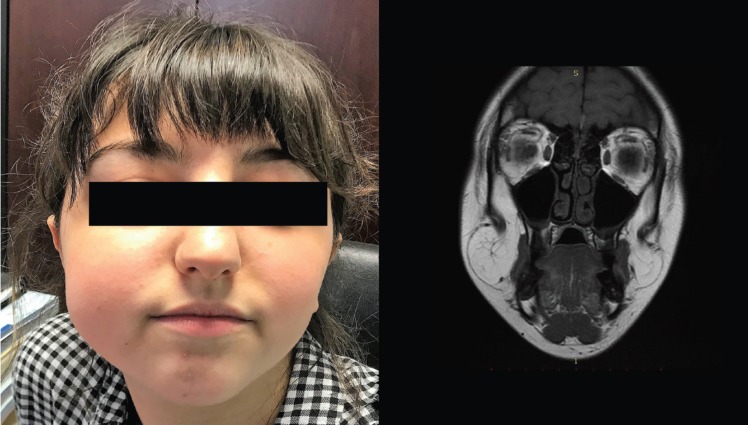
A) Preoperative photograph showing the asymmetry of the face. B) MRI imaging of the bilateral masses.

Clinical examination revealed a bilateral, soft, mobile and painless mass into the soft tissue. The mass on the right side was much larger, thus causing asymmetry (Fig. 1A).

Imaging with MRI scan showed two round-shaped, finely encapsulated masses of adipose tissue connected to the buccal fat pad (Fig. 1B). These MRI findings, in combination with the clinical examination, match the description of the buccal fat pad lipoma.

Surgery was conducted with the intraoral approach under general anesthesia, first on the right and subsequently on the left side, following the same procedure.

**Figure 2:**
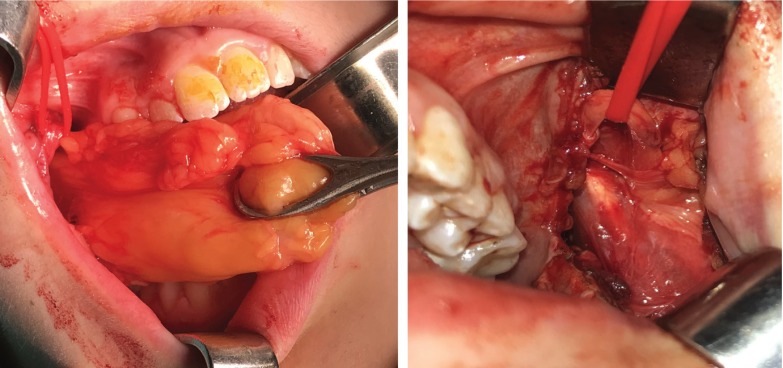
A) The right side during the surgery showing removal of the lipoma.B) The left side during the surgery.

**Figure 3:**
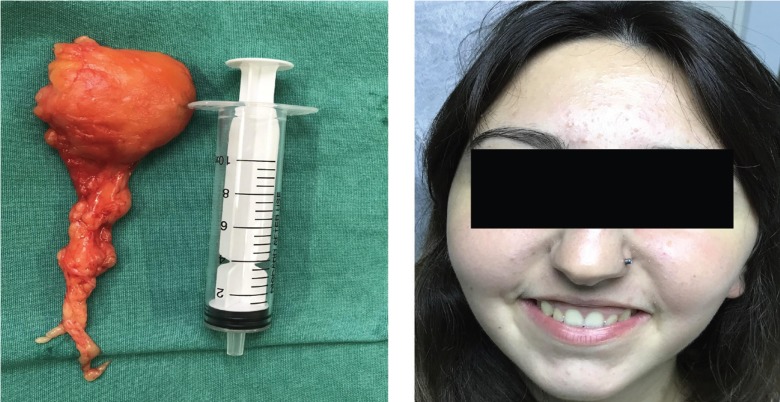
A) The lipoma of the right side after removal. B) The patient two weeks after surgery.

A relatively transverse incision was performed with a no. 15 blade through the buccal mucosa, extending from the external oblique ridge halfway up the mandibular ramus posteriorly, to approximately 5mm under the papilla of the parotid duct anteriorly, in order to recognize and protect the Stensen’s duct. A secondary incision to the buccinator muscle granted access to the lipoma. The masseter muscle, the surrounding vessels and the parotid duct were identified and protected as well. The lipoma was carefully detached from the surrounding tissues and was removed, including the well-attached larger front part of the buccal fat pad (Figs. 2A,3A). Thorough hemostasis and rinse with saline followed this procedure. The incision was carefully sutured and a drainage was placed. The same surgical technique was repeated on the left side for the removal of the smaller lipoma (Fig. 2B).

The patient was hospitalized and monitored for two days and then discharged in good general condition. Post-surgery instructions were given. Post-traumatic swelling and edema entirely disappeared two weeks after surgery (Fig. 3B).

Histological examination of the removed masses confirmed the typical image of common lipoma.

## Discussion

The buccal fat pad has plenty of functional and aesthetic roles (8). In the literature there are very few reports of lipomas attached to the buccal fad pad and, to the best of our knowledge, there is no other reported bilateral buccal fat pad. Imaging of the buccal space and its content is preferably conducted with the use of MRI. MRI is 100% specific of the common lipoma and has 100% sensitivity and 83% specificity in distinguishing a liposarcoma (3). Regarding the surgical procedure, it should be mentioned that the intraoral approach has many advantages (9). The absence of a scar following an extraoral incision is the greatest advantage that concerns patients. The intraoral approach grants enough access and is the preferable technique since MRI can be pathognomonic of the lipoma of the buccal space and can describe the benign nature of the mass.

## Conclusions

The buccal fat pad lipoma is a rare, usually asymptomatic mass that causes swelling of the buccal area. To the best of our knowledge a bilateral buccal fat pad lipoma has never been reported in the literature. Surgical attention is needed in order to secure the buccinator branch of the facial nerve, the vessels and the parotic duct in the area. Intraoral approach grants adequate access in order to remove a benign mass like lipoma and it is not necessary to adopt an extraoral approach.
